# Assessing health gradient with different equivalence scales for household income – A sensitivity analysis

**DOI:** 10.1016/j.ssmph.2021.100892

**Published:** 2021-08-04

**Authors:** Sakari Karvonen, Pasi Moisio, Kristian Vepsäläinen, Joonas Ollonqvist

**Affiliations:** Finnish Institute for Health and Welfare, Helsinki, Finland

**Keywords:** Household income, Self-reported health, Sensitivity analysis, Measurement, Health gradient, Health inequalities

## Abstract

Research on health inequalities shows considerable variation in health by socioeconomic position regardless of measurement. Income level is among the most commonly used indicators to measure the social gradient in health. The income gradient in health may, however, vary according to what aspect of health is studied but equally it may depend on how income is measured. The traditional approach of measuring income is to use household income and to modify it with consumption needs and size of the household. Our hypothesis was that the traditional picture of the income-health gradient becomes more nuanced when we use different equalization scales for household incomes than the most often used modified OECD equalization scale. More technically, we expected the steepness of the income-health gradient to change when the equivalence scale for adjusting household size and composition is altered. The data were Finnish cross-sectional 2017 data from EU-SILC (N = 9406). The primary measures were perceived health status and total household disposable annual income by household type. Ordered probit estimation using Stata package was applied to solving the function between health and income controlling for age, age-squared and gender. Respondents’ health status associated with their household type similarly to the association between personal income and household type. Those living in a single household tend to report poorer health but also tend to have lower personal income. Our main finding was that the health-income gradient becomes steeper with the larger equivalence scales, i.e., larger scale relativities, which assume bigger consumption needs for additional household members. One should be more aware of the fact that when household consumption is adjusted with conventional equivalence scales, other income-related aspects beyond consumption potential – such as social status, economic security – are also adjusted for.

## Highlights

• Selection of income equivalence scale matters not only for poverty profile but also for the distribution of poor health.
• The health-income gradient is steeper with the larger scales, which assume more consumption for other household members.
• Even relatively small changes to the scales lead to a sizeable impact on the gradient.

## 1 Introduction

Research on health inequalities shows considerable variation in health by socioeconomic position regardless of measurement (CSDH, 2008; Galobardes et al., 2007; Mackenbach, 2019). Commonly referred to as the social gradient in health, it is “a term used to describe the phenomenon whereby people who are less advantaged in terms of socioeconomic position have worse health (and shorter lives) than those who are more advantaged” (Donkin, 2014). Socioeconomic position, or SEP, is typically measured with indicators such as education, income and occupation or a combination of several indicators and the measurement is assumed to cover one or several aspects of social stratification. The social gradient in health has been shown to be very robust even though there is considerable cultural and temporal variability. However, why and how SEP influences health has raised a considerable debate over the complexity of the issue, as well as over the matter of what the best method is to measure both health and socioeconomic position in health gradients (Costa-Font & Hernández-Quevedo, 2012; Johnston et al., 2009; Mackenbach et al., 2005).

Income level is among the most common indicators to measure the social gradient in health. The income gradient in health may, however, vary according to what aspect of health is studied but equally it may depend on how income is measured. Yet, even though there is an abundance of measurements of health ranging from more objective metrices such as all-cause mortality to subjective metrices such as health complaints or self-reported health, income is most often measured only with the conventional metric of household income (Galobardes et al., 2007). How the incomes of households of different size are made comparable are rarely discussed in the income-health gradient studies, unlike in the research of income inequality and poverty (e.g., Buhmann et al., 1988; Coulter et al., 1992; Nelson, 1993; Schwarze, 2003). This is probably due to the fact that health inequalities research has primarily developed independently of research on income inequality and poverty.

Previous analyses of the income-health gradient have rested largely on the traditional approach of measuring household income, without paying much attention to sensitivity analysis. In economics and sociology, it has been widely explored how sensitive the estimates of income distribution and poverty are to the assumptions of household consumption on (e.g. Buhmann et al., 1988; De Vos & Zaidi, 1997). These studies have utilized harmonized micro-data, such as Household Budget Surveys or the Luxembourg Income Study Database allowing cross-national comparisons over a longer period of time.

The traditional measurement of household income was developed to yield the most accurate and comparable picture on the economic resources and consumption possibilities of the households of different size and composition. Under this method, a household's total disposable income is divided (adjusted) by the consumption units of the household that reflects the (assumed) consumption needs of the household. The most common consumption units are OECD and OECD modified scales, where the consumption unit of a single household is 1.0 and an additional adult adds 0.5 or 0.7 to the total consumption needs of the household. Each child adds the consumption unit by 0.3 or 0.5 correspondingly. A scale that is also used often is the square root of the size of household (OECD, 2021.).

The OECD consumption scales are based on research of household consumption, but in the end, they are conventions of the research field (Atkinson, 1998). It is well known that relatively small alterations in the consumption scales may change the estimates of income inequality and poverty substantially (e.g. De Vos & Zaidi, 1997). In the following, we will study whether adjusting the household income with different equivalence scales changes the picture of the health gradient. One of the aims of the study is to open discussion on how one should interpret the link between household income level (per consumption unit) and the personal (self-reported) health of the respondents in health inequality studies in general.

### 1.1 Research question

Broadly speaking, our aim is to reconcile the two research traditions, health inequalities research and research on income inequality and poverty. Our research hypothesis is that the traditional picture of the income-health gradient becomes more nuanced when we use different equalization scales for household incomes than the most often used modified OECD equalization scale. More technically, we expect the steepness of the income-health gradient to change when the equivalence scale for adjusting household size and composition is altered. This assumption is further elaborated upon below.

Our hypothesis is based on three observations from previous studies. The combined effect of these mechanisms on the income-health gradient is, however, difficult to assess. First, the way that household incomes are made comparable between households of different sizes and composition affects the relative position and order of these households in the income distribution (Coulter at al. 1992). Dividing household incomes with a larger equivalence scale leads to those living in a larger household to move lower on the income distribution scale, as compared to single and smaller households. However, changing the equivalence scale does not just change the rank order of households in the income distribution, it affects the estimates of inequality in a more complex manner given that the equivalence scale also modifies the overall average incomes. Using, e.g., two-parameter equivalence scales like the OECD scale that have separate weights for adults and children, the observed inequality increases along with the weight assigned to children and decreases with the weight given to adults (Figini, 1998). Second, due to the fact that personal health has a clear association with the household type and size, single households typically report lower health status than respondents living in a larger family (e.g. Hughes & Waite, 2002; Koskinen et al., 2007). Third, there is the obvious discrepancy between the fact that health is measured at the individual level while incomes are measured at the household level. In household surveys, often only one person is interviewed and health-related questions are asked of her/him directly. Analyzing the income-health gradient in a setting like this will produce data that represents the household population, where people living in a single household are overrepresented compared to the population.

## 2 Method and data

The data is Finnish cross-sectional 2017 data from the Statistics on Income and Living Conditions EU-SILC (EUROSTAT, 2021). The Finnish data is used as a pilot data for sensitivity analyses due to its good quality and reliable register-based income variables. EU-SILC has been carried out every year since 2004 among people 16 years of age or older. Data collection was conducted by Statistics Finland by means of computer-assisted interviews and mainly as telephone interviews. The interview language was either Finnish, Swedish or English depending on the preference of the interviewee. Those without a permanent address, those residing in institutional homes or permanently abroad as well as asylum seekers and those temporarily residing in Finland were excluded from the target population. After these exclusions and removals due to oversampling, the net sample in 2017 was 12 911 households. The Finnish data used here contains 9406 respondents to a household survey and their households’ members, representing the national population (OSF, 2018.). Health was measured with a question on perceived health: “How is your health in general? Is it” with five response options (1) very good, (2) good, (3) fair, (4) bad or (5) very bad? Health information was only requested from the interviewee. (Also see Tervola et al., 2021; van der Zwan & de Beer, 2021). Household income was measured with the total (previous year) disposable annual household income. Information on all incomes is derived from administrative registries and thus suffers from no recall bias. Equalized household income is annual household income divided by the consumption units of the household, described as the selected equivalence scale.

In Table 1, the descriptive figures of the data are shown. These are the respondents’ perceived health and total household disposable (net) annual income by household type. Apart from elderly couples, single-person households report lower health compared to the respondents from larger households. Of those under 65 years old living in one person households, 22% reported their health to be very good, and 52% good. Respective figures for those 65 years or older were 6% and 37%. Those living in two-adult households reported better health status compared to the single households of the same age, especially among those 65 years and older. However, clearly the highest perceived health was reported by those living in larger households with two adults and children. For instance, of those living in a household with two adults and two children, 31% reported their health to be very good, and 57% reported it to be good.

**Table 1:** Perceived health and household income by household type. EU-SILC data for Finland 2017 (N = 9406).

Household type	General health					Total %	Total N	Annual net household income (€)		
	Very good	Good	Fair	Bad	Very bad			Mean	Median	Std. Deviation
Single-person household under 65 years	22.0	51.5	20.8	5.5	0.4	100.0	1 648	21 987	19 645	15 023
Single-person household >= 65 years	6.3	37.3	43.5	11.8	1.2	100.0	773	20 670	17 695	15 604
2 adults, no dependent children, both adults under 65 years old	22.6	53.5	20.3	3.1	0.5	100.0	2 096	54 350	49 431	31 161
2 adults, no dependent children, at least one adult >=65 years old	11.5	47.6	33.7	6.4	0.8	100.0	1 690	44 982	38 586	24 644
Other households without dependent children	16.1	51.4	26.4	5.8	0.3	100.0	329	65 249	61 498	26 029
Single-parent household, one or more dependent children	25.9	52.4	17.2	3.4	1.0	100.0	290	35 825	32 584	18 116
2 adults, one dependent child	27.5	54.7	16.0	1.6	0.2	100.0	821	59 522	53 753	29 277
2 adults, two dependent children	30.9	57.0	10.4	1.6	0.1	100.0	1 043	68 246	60 677	35 677
2 adults, three or more dependent children	27.1	58.4	13.0	1.1	0.4	100.0	539	71 514	62 972	38 760
Other households with dependent children	27.1	55.9	16.9	0.0	0.0	100.0	177	77 778	74 209	28 519
Total %	20.7	51.5	22.8	4.5	0.5	100.0				
Total N	1 948	4 841	2 148	421	48	9 406	9 406			
Total								56 272	51 084	33 992

The right-hand columns of Table 1 show that the annual net household income in two- adult households is more than twice that of single-person households. This suggests that there is a selection process according to household type so that living in a one-person household predicts lower personal income and also poorer perceived health. As was discussed earlier, change in equivalization scale for adjusting household incomes will affect larger households more, change the rank order of income distribution and also influence estimates of income inequality (see Coulter at al. 1992). Since living in a larger household predicts both better perceived health and more income, the modification of equivalence scale is likely to have complex effects on the income-health gradient. The relationship between perceived health and equalized household incomes can be described as the equation EQ (1):

$$H_{t}=\theta \;\ln\left(INCOME_{t}\right)+\beta X+\varepsilon_{t}$$

where $H_{t}$ is an individual's self-assessed health, $INCOME_{t}$ is the equalized household income and the vector X describes the additional individual controls (including constants). Equalized household disposable income is defined as:

$$INCOME_{t}=sum\;of\;personal\;disposable\;incomes\;of\;the\;household/\left(1+A\left(Number\;of\;adults\;-1\right)+B\left(Number\;of\;children\right)\right)$$

We estimate EQ (1) by using ordered probit estimation (e.g., Fletcher & Wolfe, 2014) and perform it separately for each possible value of A∈[0,0,1,...,1] and B ∈[0,0.1,…,1]. Estimations are carried out using the oprobit Stata package. In the estimation, we use household-level weights and the additional controls used are age, age-squared and gender. Our interest is in studying how the parameter θ and the marginal effects of income vary depending on the choice of equivalence scale, in other words, how the health-income gradient varies depending on the choice of equivalence scale. We focus primarily on the two-parameter OECD scales that provided separate weights for adults and children, but some descriptives are also provided for one-parameter scales like the square root scale (OECD, 2021).

## 3 Analysis

We can start by looking into how the selection of equivalence scale changes the association between income groups and health. Table 2a presents perceived health (of the respondent) by income quintile when calculated either using the “old” OECD, the new OECD modified or the square root equivalence scale. Income quintiles are estimated from the total sample, hence from the equalized incomes of all members of a household. The selection of the scale seems to have a rather small influence on the distribution of perceived health by income quintile. With the OECD scale, 8 per cent of those in the lowest income quintile report their health to be bad or very bad, 7 per cent when using the OECD modified scale and 8 per cent when the square root scale is used. However, the marginal distribution column – which reports the number of cases in each income group - indicates that the less we adjust household incomes with the equivalence scale, the relatively more respondents are classified in the lowest income quintile, i.e., there are clearly more cases in the lowest income group when using the OECD scale than the modified scale. This is simply due to the income quintiles being calculated using the population sample data that includes both the respondents and their family members. As Table 2a includes only one respondent from each household, in the data, single-person households are “overrepresented” compared to respondents from larger households.

**Table 2a:** Perceived health by income quintiles with OECD and OECD-modified equivalence scales. EU-SILC data for Finland 2017 (N = 9227).

Income percentile OECD	Very good (%)	Good (%)	Fair (%)	Bad (%)	Very bad (%)	All (%)	All (N)
I (lowest)	16	45	31	7	1	100	2316
II	17	50	27	6	1	100	1732
III	20	54	22	4	1	100	1681
IV	23	56	17	3	0	100	1709
V (highest)	28	55	15	2	0	100	1789
All (%)	21	51	23	5	1	100	9227
All (N)	1900	4737	2123	420	47		9227
							
Income percentile OECD modified	Very good (%)	Good (%)	Fair (%)	Bad (%)	Very bad (%)	All (%)	All (N)
I (lowest)	17	46	29	6	1	100	1961
II	17	49	27	6	1	100	1732
III	19	51	25	5	1	100	1715
IV	22	56	19	3	0	100	1852
V (highest)	27	55	15	2	0	100	1967
All (%)	21	51	23	5	1	100	9227
All (N)	1900	4737	2123	420	47		9227
							
Income percentile square root	Very good (%)	Good (%)	Fair (%)	Bad (%)	Very bad (%)	All (%)	All (N)
I (lowest)	16	45	31	7	1	100	2457
II	17	50	28	5	1	100	1767
III	20	54	21	4	1	100	1639
IV	23	57	17	3	0	100	1655
V (highest)	29	54	14	2	0	100	1709
All (%)	21	51	23	5	1	100	2457
All (N)	1900	4737	2123	420	47	9227	1900

Table 2b presents the same figures, but now each health category is distributed into the income quintiles. Using the “old” OECD scale, one can observe that 41–45 per cent of those 467 respondents who reported their health to be bad or very bad are grouped into the lowest income quintile, while using the OECD modified scale the respective proportion is 30–40 per cent, and with the square root scale 43–49 per cent. So even with relatively modest changes in the equivalence scale such as these, the picture of how many of those report poor health and also have low income is considerably different. It appears further that the stronger one adjusts for household income by size, the smaller the proportion of those reporting (very) bad health are classified as having low income.

**Table 2b:** Perceived health by income quintiles with OECD and OECD modified equivalence scales. EU-SILC data for Finland 2017 (N = 9227).

Income percentile OECD	Very good (%)	Good (%)	Fair (%)	Bad (%)	Very bad (%)	All (%)
I (lowest)	19	22	34	41	45	25
II	15	18	22	24	19	19
III	18	19	17	14	19	18
IV	21	20	14	13	9	19
V (highest)	27	21	12	9	9	19
All (%)	100	100	100	100	100	100
All (N)	1900	4737	2123	420	47	9227
						
Income percentile OECD modified	Very good (%)	Good (%)	Fair (%)	Bad (%)	Very bad (%)	All (%)
I (lowest)	18	19	27	30	40	21
II	16	18	22	25	21	19
III	17	19	20	19	19	19
IV	21	22	16	15	9	20
V (highest)	28	23	14	11	11	21
All (%)	100	100	100	100	100	100
All (N)	1900	4737	2123	420	47	9227
						
Income percentile square root	Very good (%)	Good (%)	Fair (%)	Bad (%)	Very bad (%)	All (%)
I (lowest)	21	23	36	43	49	27
II	16	19	23	23	17	19
III	17	19	16	15	17	18
IV	20	20	13	11	11	18
V (highest)	26	20	12	8	6	19
All (%)	100	100	100	100	100	100
All (N)	1900	4737	2123	420	47	9227

**Fig. 1:**
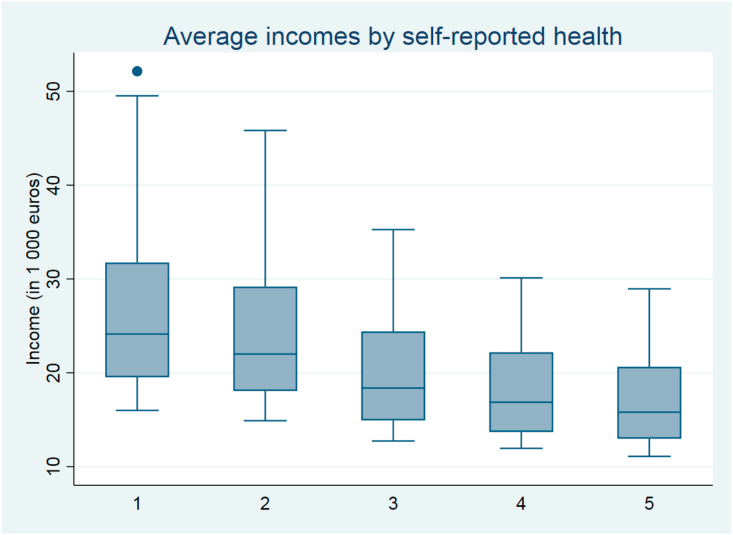
Average disposable income according to perceived health categories (very good = 1, very bad = 5) using 121 equivalence scale weights (N = 9227).

We can analyze the effect of the equivalization scale on the health-income gradient in a more detailed way by using ordered probit estimation separately for each possible scale. Allowing the consumption units of additional adults (A) and children (B) to vary between 0.0 and 1.0 with 0.1 intervals results in 121 different equivalization scales, and 121 different income-health gradients displaying the variation in the association between respondent's perceived health and household of equivalized net annual income. Fig. 1 illustrates how the average disposable income of each group which self-reported health varies depending on which adult and child weight is used in the equivalence scale. Those reporting their health to be very good (=1) have both the highest median and the largest deviation in equivalised incomes.

**Fig. 2:**
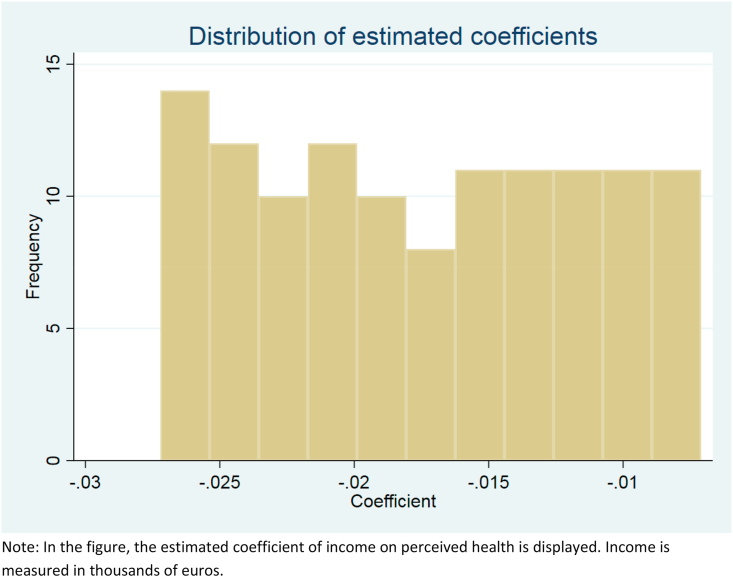
Distribution of estimated coefficients between 121 combinations of adults’ and children's weights in the equivalence scale. Note: In the figure, the estimated coefficient of income on perceived health is displayed. Income is measured in thousands of euros.

Fig. 2 shows how the estimated coefficients vary between the 121 combinations of adults’ (A) and children's (B) weights in the equivalence scale. There clearly is variation according to the choice of the scale. On the far left, the coefficients with higher weights (i.e., A and B near 1) are estimated and on the far right those coefficients are estimated with low weights (i.e. A and B are near 0). This indicates that a one thousand euro increase in equivalized household income increases the expected health more with equivalence scales with higher weights. All the coefficients are also highly significant.

**Fig. 3:**
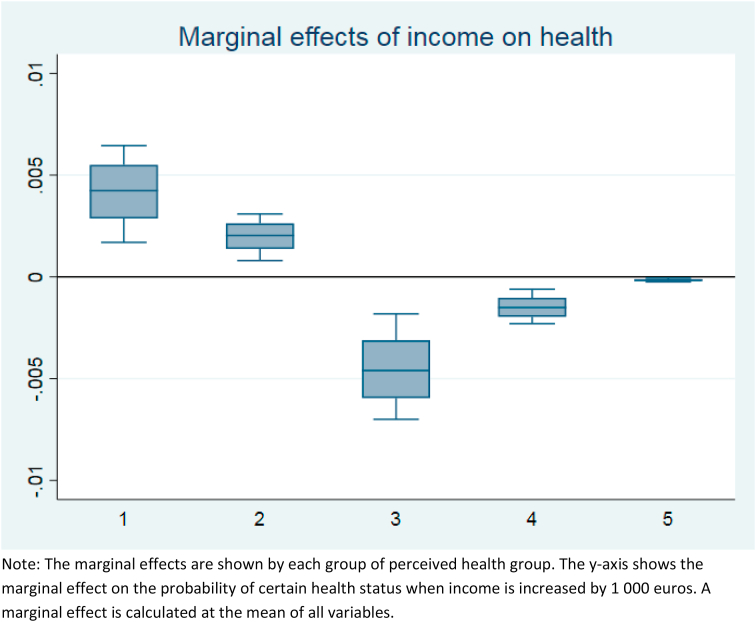
The marginal effects of income on the probability of each category of perceived health status controlling for age, age-squared and gender. A total of 112 estimations of equivalence scale. Note: The marginal effects are shown by each group of perceived health group. The y-axis shows the marginal effect on the probability of certain health status when income is increased by 1 000 euros. A marginal effect is calculated at the mean of all variables.

Furthermore, Fig. 3 shows the marginal effects of an increase in income on the probability of each health status. These are calculated as the mean of all control variables. The variation in the marginal effect is larger with good or very good health and nearly non-existent with bad health. With the larger weights (i.e., with larger A and B), the magnitude of the marginal effect is larger whereas with smaller weights the marginal effect is closer to zero. This means that the one thousand euro increase in income increases the probability of very good health and this effect is stronger the heavier adults’ (A) and children's (B) weights are in the equivalence scale. In other words, the health-income gradient is steeper with the larger equivalence scales (or larger scale relativities e.g. Coulter at al. 1992) that assume bigger consumption needs for additional household members.^1^

Finally, Table 3 shows the estimated coefficients and the marginal effects for selected equivalence scales. We can see that the steepest gradient slope, according to the marginal effects, is produced by the per-capita income scale (A,B = 1). On the other end, the least steep gradient is produced by the household income (A,B = 0). All other gradients fall somewhere between these two extremes. Comparing the two patterns illustrates that the more one weighs the consumption needs of additional household members, the steeper the income-health gradient becomes. Even with a rather slight alteration in the consumption weight, e.g., between the old and the new OECD scales, one can see a clear difference in the steepness of the gradient. As a result of this change, the “old” OECD scale produces a steeper income-health gradient than the modified OECD scale.

**Table 3:** Estimation results for selected equivalence scales.

	Coefficient	Marginal effects				
		Very good	Good	Fair	Bad	Very bad
		Health	health	health	health	health
Household income (A = 0 and B = 0)	−0.0072082	0.0017112	0.0008173	−0.0018522	−0.000611	−0.0000654
	(0.0003992)	(0.0000965)	(0.0000594)	(0.0001071)	(0.0000397)	(9.27e-06)
						
Mod. OECD scale	−0.0180721	0.0042819	0.0020538	−0.0046501	−0.0015241	−0.0001615
(A = 0.5 and B = 0.3)	(0.0009646)	(0.0002332)	(0.0001459)	(0.00026)	(0.0000966)	(0.0000228)
						
OECD scale	−0.0218552	0.0051813	0.0024818	−0.005622	−0.0018453	−0.0001958
(A = 0.7 and B = 0.5)	(0.0011799)	(0.0002853)	(0.0001775)	(0.0003177)	(0.0001179)	(0.0000277)
						
Square root	−0.0127534	0.003023	0.0014485	−0.0032811	−0.0010763	−0.0001141
	(0.000686)	(0.0001659)	(0.0001034)	(0.0001848)	(0.0000686)	(0.0000161)
						
Per-capita income (A = 1 and B = 1)	−0.0268232	0.0063686	0.0030403	−0.0068947	−0.0022723	−0.000242
	(0.0014928)	(0.0003611)	(0.0002214)	(0.0004007)	(0.0001483)	(0.0000344)

**NOTE:** Standard errors in parentheses. Estimated effect of income (measured as 1000 euros) on self-reported health with ordered probit. The marginal effects are calculated at the mean.

## 4 Discussion

Use of equivalence scales is the customary way to make the incomes of households with different size and composition comparable. Previous research has shown that the effects of selection of equivalance scale results on inequality and poverty estimates that are difficult to predict (e.g. Coulter et al., 1992). When household equivalized incomes are contrasted with a personal health, the effect of scale on the income- health gradient is even more complex. Respondents’ health associates with their household type similarly to the association between personal income and household type. As we have shown, those living in single households tend to report poorer health but also tend to have both lower household and personal income.

Generally, our main finding was that the health-income gradient becomes steeper with the larger equivalence scales, i.e., larger scale relativities, which assume bigger consumption needs for additional household members. The largest variation between scales was found among those with fair or very good health, while the variation is nearly non-existent among those with bad health. Some variation between scales was found among those with good or bad health. The effect of equivalence scale relativity, or weight, on the income-health gradient depends on the coefficient of A and B, which resembles the findings on inequality and poverty metrices (e.g. Jenkins & Cowell, 1994).

Further, it appears that the stronger one adjusts household income by its size (i.e. consumption needs) in the equivalence scale, the smaller the proportion of those reporting bad health becomes classified as having low income. Interestingly, the change in the “income profile” of those reporting poor health is in many ways similar to the change in the distribution of relative poverty among the elderly and children when moving from OECD to OECD modified scale. Changing to the lighter OECD modified scale increased the poverty rates of single households compared to the poverty rates among larger households, even though the overall poverty rates remain more or less the same. Consequently, when the modified scale was adopted for statistical use, the changing of poverty rates among children and elderly single-person households is likely to have had policy implications (see Atkinson, 1998; Burkhauser et al., 1996; de Vos; Zaidi 1997).

Our results provoke a further question of what is it exactly that the household equivalent incomes are assumed to measure in relation to the income-health gradient? The equivalence scales were developed for comparing the economic resources and material consumption needs between households of different size and composition. They are expected to describe "household welfare" in terms of a material standard of living that is presumed to be shared equally by all household members (see Nelson, 1993).

The income-health gradient represents the common practice of comparing the household equivalent income with the respondent's *personal* health status. We have compared the perceived health of household respondents to the equalized income and income quintiles that are calculated using the whole sample data that includes both the respondents and their family members. This discrepancy leads to an atomistic data structure, where, e.g., domestic economic practices are ignored. In addition to the fact that the single person-households are “overrepresented” in comparison with respondents from larger households in the data, in the latter case, the possibility of unequal consumption and inequalities in financial organization of a household are ignored. However, studies have shown that finances are often the source of intra-household inequality (e.g., Vogler & Pahl, 1994).

Equally, when household consumption needs are adjusted with conventional equivalence scales, one also “adjusts” for many other income-related aspects beyond consumption potential – such as social status or economic security. Due to equalizing, e.g., two well-paid professionals with three or four children may end up having the same equivalent household income as a young single person with an average paid temporary job. Furthermore, to the extent that income level associates with other measures of SEP, such as education, equalization may control for some health-related aspects: compliance with health information, health-enhancing/compromising behaviors and even biological health potential represented e.g., by height. Sociological studies of health have shown that higher income may involve adopting distinctive behavioral patterns that lead to better health (Bourdieu, 1984; Cockerham et al., 1997).

In light of consumption and economic resources, comparing equivalent household incomes makes sense, but when applied to the income-health gradient, one should be wary of generalizing the association beyond this. One option could be to develop an equivalence scale that also takes into account the other factors that go beyond consumption, such as using a household's subjective evaluations on their income or economic status to estimate equivalence scales (see Schwarze, 2003).

This study is based on cross-sectional data derived from the Statistics on Income and Living Conditions EU-SILC (EUROSTAT, 2021). The facts that data are cross-sectional and self-reported by only one respondent per household are limitations of the study. However, the validity of the main outcome measure is supported by the fact that they are used commonly in studies analyzing the income-health gradient (e.g. Aittomäki et al., 2010; Feng et al., 2012; Mackenbach et al., 2005). Further, it has been shown to possess high predictive validity of other medical outcomes (e.g. Idler & Benyamini, 1997). Nevertheless, no causal inferences should be made based on these associations. This was not, however, our interest to begin with. Moreover, our aim here was to provide an example of the sensitivity of the gradient to the scale adjustments, not to assess the causality of the direction or the magnitude of the association. It is highly likely that the curve series observed from these data varies when applied to other data and contexts.

To conclude, this study does not imply that there is something fundamentally wrong either with how we measure health or income in the income-health gradient studies per se (also see Costa-Font & Hernández-Quevedo, 2012). Instead, we simply want to underline that comparing household income levels with personal health entails a number of assumptions that one should be more aware of. Similar sensitivity analyses are needed regarding other measures of socio-economic position as well as their mutual associations with health status. Further studies should also focus on detailing how sensitive the effects of different scales are in different contexts, countries and when using different measures of health.

## Ethical statement

Statistics on Income and Living Conditions EU-SILC data were collected under Regulation (EC) No 1177/2003 of the European Parliament and of the Council of 16 June 2003 concerning Community statistics on income and living conditions (EU-SILC).

https://eur-lex.europa.eu/legal-content/EN/TXT/?uri=CELEX:32003R1177.

## Author statement

All authors have seen and approved the version of the manuscript that is now being submitted. All authors warrant that the article is our original work, hasn't received prior publication and isn't under consideration for publication elsewhere.

## Footnotes

1 We conducted the analysis also using log of incomes and the results remain almost the same. The only exceptions are that the upper tail is concentrated closer to the median and the differences between the commonly used equivalence scales nearly vanish.
